# Supplementary material for: Multi-trait selection for drought-tolerant soybean accessions under contrasting water regimes
Source: PLoS One. 2026 Apr 2;21(4):e0344624. doi: 10.1371/journal.pone.0344624 (PMC13046122; doi:10.1371/journal.pone.0344624)
Supplement: S1 File — S1 Fig. Rainfall pattern under water-stressed and well-watered conditions. S2 Fig. Minimum and maximum temperature pattern under water-stress and well-watered conditions. S2 Table: Description of the traits measured to evaluate the soybean accessions under water stress and well-watered conditions. S3 Table: Grain yield under stress and non-stress conditions and various tolerance indices of the screened soybean accessions. S4 Table: Factor loadings, communalities, uniquenesses and predicted genetic values of the selected accessions under water-stressed conditions based on the multi-trait genotype-ideotype distance index (Bold values represent traits with high contribution to each component). S5 Table: Factor loadings, communalities, uniquenesses and predicted genetic values of the selected accessions under well-watered conditions based on the multi-trait genotype-ideotype distance index (Bold values represent traits with high contribution to each component). (ZIP) [file pone.0344624.s001.zip › Supporting information/S2 Table.docx]

**S2 Table**. Description of the traits measured to evaluate the soybean genotypes under drought stress and well-watered conditions

| Traits | Description |
| --- | --- |
| D50F | Number of days from the date of sowing until half of the plants within each plot have opened at least one flower. |
| D95M | Number of days taken from sowing till 95% of the total plants in each plot reached full maturity. |
| FB | This trait was measured by randomly uprooting two plants from each plot at 50 days after sowing (DAS). The total fresh weight of the uprooted plants was then recorded using a digital balance. |
| PH | It was measured, in centimetres (cm), from the ground level to the tip of the plant at the maturity stage. Measurements were taken from five randomly selected plants per plot, using a meter ruler. |
| NPP | The total count of pods on five randomly selected plants in each plot at maturity. |
| NSPP | The total number of seeds from five randomly chosen pods on each of five randomly sampled plants per plot. |
| LS | The lodging score was conducted based on the IITA descriptors at maturity (R8) when 95% of the pods had reached their mature colour. The scale used was as follows: 1 = all plants are erect, 2 = 25% of plants are lodged, 3 = 50% of the plants are lodged, 4 = 75% of plants are lodged, and 5 = all plants are lodged. |
| HSW | Following threshing and drying, a sample of one hundred seeds from each genotype per replicate were counted and weighed in grams using a digital balance. |
| GY | After threshing and drying, the total weight of the harvested seeds was recorded in grams (TSW). GY was estimated using this formula:  GY (kg/ha) = [10,000 x (TSW/1000)]/A  Where: A is the plot area = number of rows x row spacing x row length. |
