# Supplementary material for: Multi-trait selection for drought-tolerant soybean accessions under contrasting water regimes
Source: PLoS One. 2026 Apr 2;21(4):e0344624. doi: 10.1371/journal.pone.0344624 (PMC13046122; doi:10.1371/journal.pone.0344624)
Supplement: S1 File — S1 Fig. Rainfall pattern under water-stressed and well-watered conditions. S2 Fig. Minimum and maximum temperature pattern under water-stress and well-watered conditions. S2 Table: Description of the traits measured to evaluate the soybean accessions under water stress and well-watered conditions. S3 Table: Grain yield under stress and non-stress conditions and various tolerance indices of the screened soybean accessions. S4 Table: Factor loadings, communalities, uniquenesses and predicted genetic values of the selected accessions under water-stressed conditions based on the multi-trait genotype-ideotype distance index (Bold values represent traits with high contribution to each component). S5 Table: Factor loadings, communalities, uniquenesses and predicted genetic values of the selected accessions under well-watered conditions based on the multi-trait genotype-ideotype distance index (Bold values represent traits with high contribution to each component). (ZIP) [file pone.0344624.s001.zip › Supporting information/S3 Table.docx]

**S3 Table:** Grain yield and drought tolerance indices values of the soybean genotypes screened under stress and non-stress conditions.

| Genotypes | Ys | Yp | STI | GMP | MP | HM | TOL | YSI | YI | SSI |
| --- | --- | --- | --- | --- | --- | --- | --- | --- | --- | --- |
| SY001 | 976.70 | 2664.94 | 0.61 | 1588.91 | 1820.82 | 1395.45 | 1688.24 | 0.39 | 1.14 | 1.01 |
| SY002 | 818.42 | 2754.27 | 0.41 | 1478.61 | 1786.34 | 1236.08 | 1935.86 | 0.29 | 0.91 | 1.18 |
| SY003 | 755.37 | 2402.14 | 0.32 | 1331.04 | 1578.76 | 1130.54 | 1646.77 | 0.30 | 0.83 | 1.16 |
| SY004 | 494.47 | 2871.39 | 0.25 | 1190.02 | 1682.93 | 842.54 | 2376.91 | 0.18 | 0.56 | 1.34 |
| SY005 | 1597.32 | 2314.22 | 0.71 | 1922.64 | 1955.77 | 1890.07 | 716.90 | 0.69 | 1.83 | 0.51 |
| SY006 | 621.56 | 3049.21 | 0.38 | 1304.20 | 1835.39 | 968.60 | 2427.65 | 0.26 | 0.77 | 1.19 |
| SY007 | 1122.84 | 2060.38 | 0.46 | 1495.57 | 1591.61 | 1408.76 | 937.55 | 0.61 | 1.35 | 0.60 |
| SY008 | 457.39 | 2921.07 | 0.37 | 1141.47 | 1689.23 | 781.97 | 2463.69 | 0.16 | 0.57 | 1.37 |
| SY009 | 985.46 | 3090.81 | 0.59 | 1739.97 | 2038.13 | 1487.71 | 2105.35 | 0.33 | 1.15 | 1.08 |
| SY010 | 1680.75 | 2657.33 | 0.84 | 2106.02 | 2169.04 | 2045.44 | 976.58 | 0.67 | 1.95 | 0.53 |
| SY011 | 1140.39 | 2365.48 | 0.55 | 1637.43 | 1752.93 | 1530.61 | 1225.10 | 0.50 | 1.34 | 0.81 |
| SY012 | 539.22 | 2458.22 | 0.24 | 1144.41 | 1498.72 | 877.85 | 1919.00 | 0.24 | 0.62 | 1.23 |
| SY013 | 1275.67 | 2333.61 | 0.55 | 1721.89 | 1804.64 | 1643.57 | 1057.94 | 0.53 | 1.44 | 0.78 |
| SY014 | 504.35 | 1833.49 | 0.15 | 930.78 | 1168.92 | 751.81 | 1329.14 | 0.41 | 0.58 | 0.93 |
| SY015 | 512.62 | 3651.21 | 0.37 | 1112.00 | 2081.92 | 759.29 | 3138.59 | 0.23 | 0.68 | 1.24 |
| SY016 | 456.55 | 1955.17 | 0.16 | 941.25 | 1205.86 | 736.96 | 1498.62 | 0.22 | 0.51 | 1.28 |
| SY017 | 748.36 | 2315.32 | 0.35 | 1279.36 | 1531.84 | 1083.20 | 1566.97 | 0.38 | 0.91 | 1.00 |
| SY018 | 501.20 | 1633.28 | 0.21 | 875.29 | 1067.24 | 732.18 | 1132.08 | 0.33 | 0.63 | 1.08 |
| SY019 | 837.29 | 1668.92 | 0.28 | 1125.07 | 1253.11 | 1019.65 | 831.63 | 0.63 | 1.04 | 0.56 |
| SY020 | 777.55 | 2828.96 | 0.47 | 1462.30 | 1803.25 | 1196.28 | 2051.40 | 0.30 | 0.94 | 1.14 |
| SY021 | 980.70 | 2456.96 | 0.51 | 1549.88 | 1718.83 | 1398.35 | 1476.27 | 0.40 | 1.14 | 0.99 |
| SY022 | 1186.03 | 2281.63 | 0.48 | 1636.56 | 1733.83 | 1546.01 | 1095.60 | 0.57 | 1.36 | 0.68 |
| SY023 | 895.23 | 2164.20 | 0.37 | 1379.54 | 1529.72 | 1247.47 | 1268.97 | 0.46 | 1.05 | 0.87 |
| SY024 | 567.75 | 1541.72 | 0.20 | 916.95 | 1054.73 | 803.95 | 973.97 | 0.40 | 0.70 | 0.97 |
| SY025 | 790.36 | 2540.48 | 0.45 | 1416.74 | 1665.42 | 1205.31 | 1750.12 | 0.31 | 0.94 | 1.12 |
| SY026 | 612.29 | 2112.00 | 0.33 | 1128.71 | 1362.15 | 939.60 | 1499.71 | 0.29 | 0.75 | 1.15 |
| SY027 | 1075.55 | 3136.14 | 0.60 | 1824.78 | 2105.85 | 1585.47 | 2060.59 | 0.38 | 1.24 | 1.00 |
| SY028 | 610.32 | 2692.66 | 0.30 | 1275.69 | 1651.49 | 989.44 | 2082.34 | 0.22 | 0.69 | 1.28 |
| SY029 | 520.40 | 1854.61 | 0.23 | 976.16 | 1187.51 | 805.67 | 1334.20 | 0.29 | 0.63 | 1.16 |
| SY030 | 603.28 | 2402.10 | 0.27 | 1194.83 | 1502.69 | 954.78 | 1798.82 | 0.28 | 0.70 | 1.18 |
| SY031 | 683.26 | 2588.59 | 0.33 | 1321.73 | 1635.93 | 1072.65 | 1905.33 | 0.25 | 0.77 | 1.23 |
| SY032 | 1657.95 | 2679.28 | 0.74 | 2083.44 | 2168.62 | 2006.40 | 1021.33 | 0.54 | 1.77 | 0.77 |
| SY033 | 998.72 | 2817.83 | 0.50 | 1676.33 | 1908.27 | 1473.05 | 1819.11 | 0.37 | 1.13 | 1.03 |
| SY034 | 538.44 | 1970.93 | 0.28 | 1025.53 | 1254.69 | 840.74 | 1432.49 | 0.27 | 0.66 | 1.18 |
| SY035 | 1116.01 | 2955.08 | 0.57 | 1811.65 | 2035.54 | 1613.79 | 1839.08 | 0.41 | 1.26 | 0.96 |
| SY036 | 1210.83 | 3634.02 | 0.76 | 2055.39 | 2422.42 | 1757.69 | 2423.19 | 0.42 | 1.41 | 0.92 |
| SY037 | 779.80 | 2461.96 | 0.37 | 1373.71 | 1620.88 | 1169.22 | 1682.16 | 0.35 | 0.92 | 1.06 |
| SY038 | 619.93 | 1373.07 | 0.15 | 922.35 | 996.50 | 853.78 | 753.14 | 0.46 | 0.70 | 0.88 |
| SY039 | 426.52 | 1905.61 | 0.16 | 868.32 | 1166.07 | 664.44 | 1479.09 | 0.28 | 0.52 | 1.16 |
| SY040 | 359.32 | 2408.00 | 0.26 | 876.64 | 1383.66 | 596.49 | 2048.69 | 0.15 | 0.47 | 1.37 |
| SY041 | 732.37 | 2047.72 | 0.28 | 1223.08 | 1390.04 | 1076.81 | 1315.35 | 0.35 | 0.83 | 1.07 |
| SY042 | 995.29 | 2015.61 | 0.38 | 1399.59 | 1505.45 | 1304.12 | 1020.32 | 0.55 | 1.18 | 0.71 |
| SY043 | 1398.92 | 2523.00 | 0.72 | 1877.86 | 1960.96 | 1798.41 | 1124.08 | 0.56 | 1.63 | 0.71 |
| SY044 | 608.82 | 1115.84 | 0.16 | 818.57 | 862.33 | 777.93 | 507.02 | 0.56 | 0.74 | 0.70 |
| SY045 | 1115.65 | 2672.89 | 0.51 | 1725.86 | 1894.27 | 1572.71 | 1557.24 | 0.43 | 1.24 | 0.92 |
| SY046 | 800.97 | 2770.30 | 0.39 | 1482.03 | 1785.63 | 1234.23 | 1969.33 | 0.27 | 0.89 | 1.20 |
| SY047 | 1426.42 | 2737.39 | 0.67 | 1972.88 | 2081.90 | 1870.29 | 1310.96 | 0.49 | 1.54 | 0.85 |
| SY048 | 1159.38 | 3264.07 | 0.68 | 1944.84 | 2211.72 | 1710.35 | 2104.69 | 0.36 | 1.31 | 1.04 |
| SY049 | 771.44 | 2044.35 | 0.30 | 1248.54 | 1407.89 | 1109.60 | 1272.91 | 0.41 | 0.90 | 0.96 |
| SY050 | 905.41 | 2326.69 | 0.42 | 1438.41 | 1616.05 | 1285.11 | 1421.29 | 0.39 | 1.03 | 1.02 |
| SY051 | 866.82 | 2542.32 | 0.37 | 1461.44 | 1704.57 | 1260.21 | 1675.50 | 0.43 | 1.00 | 0.92 |
| SY052 | 1470.70 | 2225.36 | 0.56 | 1806.05 | 1848.03 | 1765.19 | 754.66 | 0.72 | 1.62 | 0.44 |
| SY053 | 1091.32 | 2192.40 | 0.41 | 1546.73 | 1641.86 | 1457.13 | 1101.07 | 0.49 | 1.19 | 0.83 |
| SY054 | 1069.94 | 2874.61 | 0.56 | 1726.28 | 1972.27 | 1519.16 | 1804.67 | 0.44 | 1.26 | 0.89 |
| SY055 | 527.02 | 2751.47 | 0.25 | 1203.37 | 1639.24 | 883.96 | 2224.45 | 0.19 | 0.58 | 1.33 |
| SY056 | 1345.67 | 1774.71 | 0.41 | 1544.11 | 1560.19 | 1528.27 | 429.04 | 0.72 | 1.46 | 0.46 |
| SY057 | 626.39 | 2476.85 | 0.34 | 1243.52 | 1551.62 | 997.78 | 1850.46 | 0.25 | 0.73 | 1.23 |
| SY058 | 830.11 | 2149.37 | 0.37 | 1324.16 | 1489.74 | 1181.23 | 1319.26 | 0.39 | 0.95 | 1.02 |
| SY059 | 1021.37 | 3360.68 | 0.62 | 1846.43 | 2191.03 | 1559.26 | 2339.31 | 0.29 | 1.14 | 1.17 |
| SY060 | 748.39 | 2074.41 | 0.28 | 1226.37 | 1411.40 | 1071.67 | 1326.02 | 0.43 | 0.88 | 0.91 |
| SY061 | 866.37 | 3271.50 | 0.48 | 1681.37 | 2068.94 | 1367.68 | 2405.14 | 0.25 | 0.95 | 1.23 |
| SY062 | 465.60 | 2393.98 | 0.21 | 1055.38 | 1429.79 | 779.27 | 1928.38 | 0.20 | 0.53 | 1.31 |
| SY063 | 802.73 | 2176.56 | 0.32 | 1321.42 | 1489.65 | 1172.34 | 1373.84 | 0.36 | 0.90 | 1.05 |
| SY064 | 780.50 | 1760.33 | 0.29 | 1171.54 | 1270.42 | 1080.52 | 979.83 | 0.45 | 0.92 | 0.90 |
| SY065 | 1201.93 | 2075.81 | 0.46 | 1558.69 | 1638.87 | 1484.42 | 873.88 | 0.67 | 1.41 | 0.51 |
| SY066 | 849.96 | 3009.57 | 0.51 | 1576.73 | 1929.77 | 1299.05 | 2159.61 | 0.31 | 1.02 | 1.11 |
| SY067 | 850.25 | 2640.63 | 0.47 | 1497.55 | 1745.44 | 1285.25 | 1790.38 | 0.33 | 1.00 | 1.10 |
| SY068 | 736.34 | 1988.62 | 0.25 | 1204.15 | 1362.48 | 1066.10 | 1252.28 | 0.41 | 0.84 | 0.95 |
| SY069 | 344.61 | 1778.94 | 0.13 | 769.14 | 1061.77 | 566.00 | 1434.33 | 0.21 | 0.42 | 1.28 |
| SY070 | 443.03 | 850.06 | 0.09 | 611.95 | 646.54 | 579.52 | 407.03 | 0.53 | 0.52 | 0.78 |
| SY071 | 744.28 | 1264.92 | 0.30 | 954.75 | 1004.60 | 910.16 | 520.64 | 0.56 | 0.95 | 0.69 |
| SY072 | 461.70 | 1569.59 | 0.18 | 637.95 | 1015.65 | 527.40 | 1107.88 | 0.42 | 0.63 | 0.90 |
| SY073 | 396.94 | 1400.56 | 0.11 | 744.15 | 898.75 | 616.92 | 1003.62 | 0.28 | 0.45 | 1.19 |
| SY074 | 597.09 | 3074.37 | 0.33 | 1300.67 | 1835.73 | 952.04 | 2477.28 | 0.26 | 0.72 | 1.19 |
| SY075 | 1207.03 | 2797.45 | 0.70 | 1835.98 | 2002.24 | 1683.97 | 1590.42 | 0.44 | 1.42 | 0.91 |
| SY076 | 525.36 | 1636.50 | 0.17 | 894.74 | 1080.93 | 755.58 | 1111.14 | 0.35 | 0.59 | 1.09 |
| SY077 | 659.47 | 2308.03 | 0.29 | 1227.62 | 1483.75 | 1018.62 | 1648.56 | 0.31 | 0.77 | 1.13 |
| SY078 | 1030.13 | 1869.39 | 0.36 | 1376.14 | 1449.76 | 1307.74 | 839.25 | 0.61 | 1.21 | 0.62 |
| SY079 | 926.84 | 1994.53 | 0.40 | 1355.97 | 1460.69 | 1259.64 | 1067.69 | 0.48 | 1.10 | 0.85 |
| SY080 | 1074.27 | 3310.17 | 0.62 | 1850.85 | 2192.22 | 1574.86 | 2235.90 | 0.40 | 1.26 | 0.96 |
| SY081 | 766.19 | 2003.66 | 0.25 | 1170.15 | 1384.93 | 1001.60 | 1237.46 | 0.62 | 0.92 | 0.57 |
| SY082 | 533.90 | 2359.46 | 0.21 | 1097.87 | 1446.68 | 845.31 | 1825.56 | 0.30 | 0.62 | 1.14 |
| SY083 | 911.12 | 1817.58 | 0.29 | 1283.07 | 1364.35 | 1207.30 | 906.46 | 0.54 | 1.04 | 0.74 |
| SY084 | 589.61 | 1913.62 | 0.24 | 1054.99 | 1251.62 | 892.53 | 1324.02 | 0.32 | 0.71 | 1.10 |
| SY085 | 793.43 | 1715.53 | 0.27 | 1150.25 | 1254.48 | 1058.13 | 922.10 | 0.52 | 0.95 | 0.76 |
| SY086 | 834.86 | 1962.96 | 0.35 | 1279.06 | 1398.91 | 1169.79 | 1128.10 | 0.43 | 0.98 | 0.93 |
| SY087 | 626.82 | 2200.31 | 0.32 | 1161.96 | 1413.56 | 961.42 | 1573.49 | 0.30 | 0.73 | 1.16 |
| SY088 | 588.02 | 1871.24 | 0.23 | 1048.47 | 1229.63 | 894.22 | 1283.22 | 0.32 | 0.69 | 1.11 |
| SY089 | 604.61 | 2078.80 | 0.27 | 1107.88 | 1341.71 | 921.17 | 1474.19 | 0.31 | 0.73 | 1.12 |
| SY090 | 708.04 | 2074.96 | 0.28 | 1211.59 | 1391.50 | 1055.14 | 1366.92 | 0.35 | 0.82 | 1.07 |
| SY091 | 904.46 | 1772.53 | 0.34 | 1265.13 | 1338.50 | 1196.00 | 868.07 | 0.51 | 1.05 | 0.81 |
| SY092 | 1089.60 | 2612.80 | 0.65 | 1685.28 | 1851.20 | 1534.84 | 1523.20 | 0.42 | 1.30 | 0.94 |
| SY093 | 1037.84 | 2831.97 | 0.50 | 1703.10 | 1934.91 | 1504.24 | 1794.13 | 0.33 | 1.12 | 1.10 |
| SY094 | 1564.74 | 3157.78 | 1.00 | 2222.69 | 2361.26 | 2092.29 | 1593.04 | 0.49 | 1.81 | 0.83 |
| SY095 | 1503.65 | 2510.20 | 0.68 | 1942.22 | 2006.92 | 1879.68 | 1006.55 | 0.61 | 1.71 | 0.63 |
| SY096 | 1164.29 | 2671.05 | 0.56 | 1749.13 | 1917.67 | 1598.73 | 1506.76 | 0.49 | 1.35 | 0.82 |
| SY097 | 1190.75 | 2840.74 | 0.63 | 1833.16 | 2015.75 | 1668.78 | 1649.99 | 0.44 | 1.38 | 0.90 |
| SY098 | 1370.75 | 2505.94 | 0.60 | 1849.47 | 1938.35 | 1765.21 | 1135.19 | 0.58 | 1.55 | 0.67 |
| SY099 | 1208.51 | 2842.01 | 0.65 | 1852.13 | 2025.26 | 1694.13 | 1633.51 | 0.43 | 1.39 | 0.92 |
| SY100 | 1275.08 | 2922.48 | 0.65 | 1896.62 | 2098.78 | 1720.75 | 1647.40 | 0.53 | 1.49 | 0.74 |
| SY101 | 1418.82 | 2601.32 | 0.66 | 1918.14 | 2010.07 | 1830.85 | 1182.50 | 0.57 | 1.61 | 0.69 |
| SY102 | 833.67 | 2879.91 | 0.41 | 1523.29 | 1856.79 | 1260.02 | 2046.25 | 0.36 | 0.96 | 1.02 |
| SY103 | 1050.12 | 2678.94 | 0.48 | 1670.98 | 1864.53 | 1499.34 | 1628.82 | 0.43 | 1.19 | 0.92 |
| SY104 | 1278.00 | 2726.24 | 0.60 | 1866.48 | 2002.12 | 1740.06 | 1448.24 | 0.46 | 1.40 | 0.88 |
| SY105 | 1447.65 | 2103.46 | 0.54 | 1741.57 | 1775.55 | 1708.42 | 655.81 | 0.73 | 1.65 | 0.43 |
| SY106 | 1052.02 | 1972.02 | 0.38 | 1397.00 | 1512.02 | 1295.69 | 920.01 | 0.67 | 1.26 | 0.49 |
| SY107 | 862.82 | 1953.16 | 0.40 | 1297.05 | 1407.99 | 1195.16 | 1090.34 | 0.44 | 1.04 | 0.91 |
| SY108 | 1120.34 | 1852.15 | 0.42 | 1439.25 | 1486.25 | 1393.88 | 731.81 | 0.61 | 1.31 | 0.62 |
| SY109 | 902.12 | 2111.19 | 0.37 | 1342.14 | 1506.66 | 1204.46 | 1209.08 | 0.52 | 1.09 | 0.76 |
| SY110 | 909.06 | 2494.36 | 0.50 | 1504.94 | 1701.71 | 1331.26 | 1585.30 | 0.37 | 1.08 | 1.03 |
| SY111 | 562.00 | 2008.84 | 0.19 | 1013.19 | 1285.42 | 816.59 | 1446.84 | 0.41 | 0.67 | 0.93 |
| SY112 | 1020.25 | 2383.21 | 0.47 | 1559.31 | 1701.73 | 1428.82 | 1362.96 | 0.43 | 1.17 | 0.94 |
| SY113 | 855.39 | 2130.29 | 0.33 | 1340.59 | 1492.84 | 1207.33 | 1274.90 | 0.38 | 0.95 | 1.02 |
| SY114 | 908.87 | 1979.11 | 0.33 | 1340.69 | 1443.99 | 1244.90 | 1070.24 | 0.47 | 1.03 | 0.87 |
| SY115 | 442.89 | 2372.61 | 0.26 | 1000.53 | 1407.75 | 727.67 | 1929.72 | 0.20 | 0.55 | 1.30 |
| SY116 | 914.74 | 2997.18 | 0.51 | 1655.56 | 1955.96 | 1401.40 | 2082.45 | 0.30 | 1.04 | 1.14 |
| SY117 | 1330.93 | 3216.30 | 0.78 | 2057.79 | 2273.61 | 1865.46 | 1885.36 | 0.45 | 1.54 | 0.88 |
| SY118 | 640.96 | 2039.27 | 0.25 | 1121.02 | 1340.11 | 946.52 | 1398.31 | 0.37 | 0.76 | 1.01 |
| SY119 | 1129.85 | 2597.21 | 0.55 | 1709.58 | 1863.53 | 1569.25 | 1467.36 | 0.45 | 1.31 | 0.89 |
| SY120 | 905.42 | 1255.41 | 0.28 | 1066.11 | 1080.41 | 1052.01 | 349.99 | 0.72 | 1.08 | 0.46 |
| SY121 | 945.21 | 2882.05 | 0.53 | 1590.77 | 1913.63 | 1344.47 | 1936.84 | 0.40 | 1.15 | 0.95 |
| SY122 | 792.68 | 2055.72 | 0.31 | 1268.88 | 1424.20 | 1132.93 | 1263.04 | 0.42 | 0.93 | 0.94 |
| SY123 | 716.78 | 2485.03 | 0.41 | 1280.42 | 1600.91 | 1050.59 | 1768.24 | 0.33 | 0.90 | 1.08 |
| SY124 | 1027.02 | 2488.95 | 0.54 | 1587.67 | 1757.99 | 1437.13 | 1461.93 | 0.44 | 1.23 | 0.91 |
| SY125 | 1011.67 | 2020.02 | 0.42 | 1428.60 | 1515.84 | 1346.56 | 1008.34 | 0.51 | 1.19 | 0.80 |
| SY126 | 1136.89 | 2681.29 | 0.53 | 1740.47 | 1909.09 | 1588.63 | 1544.40 | 0.40 | 1.25 | 0.99 |
| SY127 | 1145.70 | 1668.70 | 0.40 | 1372.51 | 1407.20 | 1339.24 | 523.00 | 0.73 | 1.37 | 0.42 |
| SY128 | 639.21 | 1635.97 | 0.24 | 1021.52 | 1137.59 | 917.68 | 996.76 | 0.39 | 0.75 | 1.00 |
| SY129 | 401.17 | 1611.69 | 0.12 | 745.08 | 1006.43 | 579.83 | 1210.52 | 0.35 | 0.50 | 1.04 |
| SY130 | 850.01 | 2440.41 | 0.42 | 1435.12 | 1645.21 | 1253.86 | 1590.40 | 0.36 | 1.00 | 1.03 |
| SY131 | 276.47 | 1580.69 | 0.13 | 646.50 | 928.58 | 460.64 | 1304.22 | 0.18 | 0.35 | 1.34 |
| SY132 | 252.54 | 2390.77 | 0.15 | 764.36 | 1321.66 | 451.70 | 2138.23 | 0.11 | 0.31 | 1.45 |
| SY133 | 525.88 | 1909.88 | 0.18 | 1002.14 | 1217.88 | 824.64 | 1384.00 | 0.27 | 0.60 | 1.19 |
| SY134 | 499.19 | 2258.17 | 0.28 | 1060.72 | 1378.68 | 816.72 | 1758.98 | 0.22 | 0.60 | 1.27 |
| SY135 | 404.25 | 2100.57 | 0.16 | 920.39 | 1252.41 | 677.12 | 1696.32 | 0.20 | 0.47 | 1.31 |
| SY136 | 614.78 | 2416.26 | 0.28 | 1218.76 | 1515.52 | 980.13 | 1801.48 | 0.26 | 0.71 | 1.22 |
| SY137 | 588.07 | 1460.60 | 0.15 | 898.36 | 1024.33 | 798.71 | 872.53 | 0.41 | 0.65 | 1.00 |
| SY138 | 636.67 | 2790.43 | 0.37 | 1332.79 | 1713.55 | 1036.69 | 2153.76 | 0.23 | 0.75 | 1.26 |
| SY139 | 882.53 | 2252.41 | 0.34 | 1389.44 | 1567.47 | 1236.63 | 1369.88 | 0.49 | 1.01 | 0.82 |
| SY140 | 384.44 | 2070.31 | 0.17 | 871.33 | 1227.37 | 632.08 | 1685.86 | 0.21 | 0.47 | 1.28 |
| SY141 | 994.32 | 2339.71 | 0.63 | 1444.60 | 1667.01 | 1279.60 | 1345.39 | 0.46 | 1.28 | 0.85 |
| SY142 | 1099.41 | 2648.52 | 0.55 | 1704.94 | 1873.97 | 1551.59 | 1549.11 | 0.43 | 1.27 | 0.94 |
| SY143 | 280.15 | 2133.84 | 0.15 | 768.17 | 1207.00 | 492.66 | 1853.69 | 0.13 | 0.34 | 1.41 |
| SY144 | 640.79 | 2324.23 | 0.30 | 1219.76 | 1482.51 | 1003.91 | 1683.43 | 0.28 | 0.75 | 1.18 |
| SY145 | 831.64 | 2817.42 | 0.45 | 1512.23 | 1824.53 | 1261.65 | 1985.78 | 0.33 | 0.99 | 1.08 |
| SY146 | 620.20 | 1863.71 | 0.27 | 1012.69 | 1241.96 | 852.31 | 1243.51 | 0.39 | 0.79 | 0.97 |
| SY147 | 1050.86 | 2769.24 | 0.56 | 1628.00 | 1910.05 | 1409.98 | 1718.38 | 0.49 | 1.29 | 0.80 |
| SY148 | 595.00 | 2168.17 | 0.22 | 1128.57 | 1381.58 | 926.18 | 1573.17 | 0.25 | 0.65 | 1.23 |
| SY149 | 844.31 | 2046.04 | 0.31 | 1314.27 | 1445.18 | 1195.25 | 1201.73 | 0.41 | 0.95 | 0.97 |
| SY150 | 1417.66 | 2844.43 | 0.80 | 1994.31 | 2131.04 | 1868.91 | 1426.78 | 0.53 | 1.68 | 0.75 |
