# Supplementary material for: Multi-trait selection for drought-tolerant soybean accessions under contrasting water regimes
Source: PLoS One. 2026 Apr 2;21(4):e0344624. doi: 10.1371/journal.pone.0344624 (PMC13046122; doi:10.1371/journal.pone.0344624)
Supplement: S1 File — S1 Fig. Rainfall pattern under water-stressed and well-watered conditions. S2 Fig. Minimum and maximum temperature pattern under water-stress and well-watered conditions. S2 Table: Description of the traits measured to evaluate the soybean accessions under water stress and well-watered conditions. S3 Table: Grain yield under stress and non-stress conditions and various tolerance indices of the screened soybean accessions. S4 Table: Factor loadings, communalities, uniquenesses and predicted genetic values of the selected accessions under water-stressed conditions based on the multi-trait genotype-ideotype distance index (Bold values represent traits with high contribution to each component). S5 Table: Factor loadings, communalities, uniquenesses and predicted genetic values of the selected accessions under well-watered conditions based on the multi-trait genotype-ideotype distance index (Bold values represent traits with high contribution to each component). (ZIP) [file pone.0344624.s001.zip › Supporting information/S4 Table.docx]

**S4 Table:** Factor loadings, communalities, uniquenesses and predicted genetic values of the selected genotypes under water-stressed conditions based on the multi-trait genotype-ideotype distance index (Bold values represent traits with high contribution to each component)

| Traits | FA1 | FA2 | FA3 | FA4 | Com | Uni | SD | SD (%) | | Sense | Goal |
| --- | --- | --- | --- | --- | --- | --- | --- | --- | --- | --- | --- |
| D50F | **-0.88** | -0.08 | 0.12 | -0.27 | 0.87 | 0.13 | -1.86 | -4.40 | decrease | | 100 |
| D95M | **-0.88** | 0.32 | -0.01 | 0.00 | 0.88 | 0.12 | -0.9 | -0.83 | decrease | | 100 |
| FB | 0.21 | 0.21 | **-0.83** | -0.17 | 0.81 | 0.19 | 3.9 | 10.66 | increase | | 100 |
| PH | 0.15 | **-0.70** | -0.08 | -0.25 | 0.58 | 0.42 | -3.82 | -6.11 | increase | | 0 |
| NPP | 0.12 | 0.41 | -0.05 | **0.67** | 0.63 | 0.37 | -14.18 | -11.58 | increase | | 0 |
| NSPP | -0.03 | **-0.67** | 0.03 | 0.02 | 0.45 | 0.55 | -0.08 | -3.31 | increase | | 0 |
| LS | 0.20 | 0.27 | **0.78** | -0.07 | 0.73 | 0.27 | -0.04 | -2.24 | decrease | | 100 |
| HSW | -0.10 | 0.05 | -0.11 | **-0.88** | 0.80 | 0.20 | 0.81 | 6.47 | increase | | 100 |
| GY | **0.60** | 0.49 | 0.28 | -0.02 | 0.68 | 0.32 | -279.32 | -19.18 | increase | | 0 |
| Total decrease | -7.47 | Communality average | | | 0.71 |  |  |  |  | |  |
| Total increase | -23.12 |  |  |  |  |  |  |  |  | |  |

Com = communalities, Uni = uniquenessess, SD = genetic gains
