# Supplementary material for: Multi-trait selection for drought-tolerant soybean accessions under contrasting water regimes
Source: PLoS One. 2026 Apr 2;21(4):e0344624. doi: 10.1371/journal.pone.0344624 (PMC13046122; doi:10.1371/journal.pone.0344624)
Supplement: S1 File — S1 Fig. Rainfall pattern under water-stressed and well-watered conditions. S2 Fig. Minimum and maximum temperature pattern under water-stress and well-watered conditions. S2 Table: Description of the traits measured to evaluate the soybean accessions under water stress and well-watered conditions. S3 Table: Grain yield under stress and non-stress conditions and various tolerance indices of the screened soybean accessions. S4 Table: Factor loadings, communalities, uniquenesses and predicted genetic values of the selected accessions under water-stressed conditions based on the multi-trait genotype-ideotype distance index (Bold values represent traits with high contribution to each component). S5 Table: Factor loadings, communalities, uniquenesses and predicted genetic values of the selected accessions under well-watered conditions based on the multi-trait genotype-ideotype distance index (Bold values represent traits with high contribution to each component). (ZIP) [file pone.0344624.s001.zip › Supporting information/S5 Table .docx]

**S5 Table**: Factor loadings, communalities, uniquenesses and predicted genetic values of the selected genotypes under well-watered conditions based on the multi-trait genotype-ideotype distance index (Bold values represent traits with high contribution to each component)

| Traits | FA1 | FA2 | FA3 | FA4 | Com | Uni | SD | SD (%) | Sense | Goal |
| --- | --- | --- | --- | --- | --- | --- | --- | --- | --- | --- |
| D50F | -0.27 | **0.84** | 0.20 | 0.06 | 0.82 | 0.18 | 0.83 | 2.02 | decrease | 0 |
| D95M | -0.22 | -0.24 | **-0.80** | -0.02 | 0.74 | 0.26 | 1.35 | 1.28 | decrease | 0 |
| FB | -0.27 | 0.08 | 0.15 | **0.74** | 0.65 | 0.35 | -6.62 | -6.18 | increase | 0 |
| PH | **0.89** | -0.25 | 0.00 | 0.04 | 0.85 | 0.15 | 11.44 | 13.91 | increase | 100 |
| NPP | -0.15 | -0.26 | **0.79** | 0.03 | 0.71 | 0.29 | -7.05 | -6.16 | increase | 0 |
| NSPP | **0.73** | -0.09 | 0.10 | 0.02 | 0.55 | 0.45 | 0.15 | 5.36 | increase | 100 |
| LS | 0.04 | **0.77** | -0.17 | 0.08 | 0.62 | 0.38 | -0.34 | -21.35 | decrease | 100 |
| HSW | **0.63** | 0.31 | -0.13 | -0.29 | 0.60 | 0.40 | 0.28 | 2.07 | increase | 100 |
| GY | 0.16 | 0.06 | -0.10 | **0.88** | 0.82 | 0.18 | 884.14 | 29.45 | increase | 100 |
| Total decrease | -17.98 | Communality average | | | 0.71 |  |  |  |  |  |
| Total increase | 38.45 |  |  |  |  |  |  |  |  |  |
