# Supplementary material for: Multi-trait selection for drought-tolerant soybean accessions under contrasting water regimes
Source: PLoS One. 2026 Apr 2;21(4):e0344624. doi: 10.1371/journal.pone.0344624 (PMC13046122; doi:10.1371/journal.pone.0344624)
Supplement: Attachment — Submitted filename: Review #306 PONE-D-25-34303.pdf [file pone.0344624.s002.pdf]

Reviewer comments to: PONE-D-25-34303 "Multi-Trait Selection for Drought-Tolerant Soybean Genotypes Under Contrasting Water Regimes" for PLOS ONE

Summary

This paper describes original research in which 150 soybean accessions were subjected to withholding water at two critical points during plant growth, and repeated for two years. Performance of these accessions was also measured during well-watered conditions. The goal was to identify germplasm tolerant to water deficit stress. This paper validates multi-trait selection (MGIDI) which is not a new idea, but might be unique for soybeans in Nigeria, subjected to water deficit. The authors might add several sentences to clarify the scientific novelty of this study.

In general the experiment was conducted with appropriate methods, and statistical procedures. Sufficient experimental material was used. In general, the study was described in sufficient detail, although the soil used should be described (see note 4). The study identified 30 accessions with better tolerance of water deficit, and 8 accessions that were better in a range of conditions. No single trait best described plant water stress tolerance, and required multiple traits to identify suitable accessions.

Revision of the manuscript is suggested.. with attention to correct use of 'drought' (see note 1), and 'genotype' (see note 2). Other points are noted below.

Page numbers refer to the number listed for the manuscript body (rather than the collated PDF)

Main points

1) P1 L2 (also P5 L102-104, and throughout the manuscript) Avoid using water stress and drought stress interchangeably. Drought is variation from climatic normality. Since your water deficit is applied artificially, it is better to use the term 'water stress', or 'water deficit stress' (to distinguish from flooding stress). Drought, from below average rainfall, is likely to have seasonal duration, and might vary from the short-term water deficits used in this study. Drought is also typically associated with other climatic factors such as higher temperature and/or lower humidity, as well as water deficit. In this study, short-term water deficit is a tool for determination of accession performance, and likely is related to drought tolerance, however, that was not measured in this study.
2) P1 L3 title (also P1 L16, P1 L21, P3 L70, and throughout the document). Ensure that genotype is only used to describe a single genetic identity. Avoid the colloquial use of 'genotype' to describe a 'line', or 'accession'. In most instance, 'genotype' can be replaced with 'germplasm'. Table S1 in Supplementary materials describes the germplasm being used as 'accessions'. Its not clear in this paper if the accessions were

genotypes (i.e. inbred or clonal germplasm), but for soybeans, its likely these were selections from outcrossed germplasm, comprising many genotypes. Although there are precedents for colloquial use pf 'genotype' in the literature, "accessions" should not be referred to as 'genotypes', unless these are inbred lines comprising a single genotype.

3) P3 L48-49. Revise this sentence; as overly simplistic. Drought is infinitely variable, so giving a single yield loss is misleading. Drought can reduce soybean yield by 100%!!
4) P4 L89. Describe the soil used for the study.
5) P5 L100. Presumably, the replications were whole blocks?
6) P5 L111. Clarify 'Nodumax'. Was this a commercial product. Presumably is rhizobia?

Specific points

7) P1 L18 (also P5 L105). 'Drought' is a noun, and avoid colloquial use as an adjective. Suggest to replace 'drought stress' with 'water stress'
8) P1 L78. Use past tense 'aimed'. L79. Replace 'are', with 'were'. L86. Replace 'will proved', with 'provided'
9) P3 L56. Add 'growth', i.e. '... during critical growth stages...'
10) P4 L86. Use singular 'gain' (how many 'genetic gains' are you describing here?)
11) P4 L96. Avoid the possessive term. Suggest "The climate of Ibadan is ..."
12) P5 L107. Avoid the possessive term. Suggest "plant"
13) P5 L113. Replace "and that gives a total of", with "equivalent to"
14) P5 L114-116. Use lower case 'metribuzin, and 'urea'. Check journal style, do these chemicals need their full chemical name at first mention?
15) P17 L313. Use singular 'Multi-trait'
16) P17 L316 (also L411). Suggest to replace 'Under', with "During"
17) P17 L322. Use past tense 'was'
18) P20 L389. Changes in what?
19) P20 L391. Delete 'economic'. Drought will simply reduce yield.. not need to include the term 'economic'
20) P20 L397. Replace 'like', with 'such as' (L438-439 is correct)
21) P23 L451. Use past tense 'indicated'
