# Supplementary material for: Multi-trait selection for drought-tolerant soybean accessions under contrasting water regimes
Source: PLoS One. 2026 Apr 2;21(4):e0344624. doi: 10.1371/journal.pone.0344624 (PMC13046122; doi:10.1371/journal.pone.0344624)
Supplement: Attachment — Submitted filename: General Comments_Plosone.docx [file pone.0344624.s003.docx]

**General Comments:**

The authors used multi-trait selection indices, particularly MGIDI, in combination with traditional drought tolerance indices, across two contrasting water regimes. This strengthen the screening and selection efficiency. The manuscript is generally well-structured, with a clear rationale and thoughtful statistical methods.

However, some methodological clarifications, results interpretation improvements, and stronger framing of novelty are needed before publication.

- Several grammatical errors and non-scientific phrases are present throughout.
- Intensive English language revision is required.
- Use consistent verb tense—prefer past tense for methods and results.

**Title & Abstract**

- The abstract is long and verbose. Consider tightening the language by focusing more on outcomes than method repetition.
- Mention how this research advances the field by demonstrating MGIDI's use for drought selection in tropical soybean germplasm.

**Introduction**

- A clear articulation of the knowledge gap is lacking. What specifically is not known or has not been done in similar germplasm or environments?
- Please include information that clearly stating:
  - Why previous selection strategies were insufficient.
  - What novelty MGIDI + traditional indices bring in combination.
  - Why the IITA germplasm is uniquely suitable for this kind of selection.
- A clear hypothesis is missing.

**Materials and Methods**

- There is no control/check varieties. Without benchmark genotypes, it's difficult to interpret relative performance or derive conclusions about breeding progress.
- Are the two-stage drought simulation realistic stress?
- Temperature and rainfall variations across the years/seasons are critical in drought studies.
- Add a climatic data figure comparing environmental conditions in WW and WS seasons.
- The field management description lacks clarity on soil properties, nutrient status, and irrigation uniformity. These are critical for attributing differences in genotypic response to water regime rather than confounding effects.
- Two row per plot is not experimentally viable.
- What was the kind of sprinkler irrigation system? Model? Etc.
- It’s not clearly stated whether the experimental unit was plot-based or individual plant-based for statistical analysis.
- Clarify unit of replication and randomization protocol used within blocks. Was spatial variation corrected?
- The rationale for timing and duration needs justification using prior physiological data on soybean water stress sensitivity. How did you choose these two time points?

**Statistical Analysis**

- While explained mathematically, lacks the MGIDI implementation explanation for breeders.
- Include a statement on whether model assumptions were checked and met.

**Results & Discussion**

- Grain yield (GY) had low H² under both conditions; this warrants deeper discussion, especially since it's the main selection target. Is MGIDI offset low GY heritability? This contradicts genetic gain theory unless indirect selection traits are used.
- Ensure each figure clearly labels axes, index names, and genotype IDs.
- Add a paragraph in discussion, comparing genotypes selected by MGIDI vs. traditional indices (beyond SY150). What kind of genotypes would have been missed without MGIDI?
- The discussion focuses heavily on confirming existing findings (e.g., GY drops with drought), but misses a chance to generalize implications for other tropical soybean breeding programs.
- What can breeders take away from this dataset? How could this inform early-stage breeding or genomic selection?

**Conclusion**

- Slightly descriptive.
- Add a concise final paragraph with a call to action.
- No validation is provided for selected genotypes. Without validation, conclusions on stability remain speculative.
