# Supplementary material for: Multi-trait selection for drought-tolerant soybean accessions under contrasting water regimes
Source: PLoS One. 2026 Apr 2;21(4):e0344624. doi: 10.1371/journal.pone.0344624 (PMC13046122; doi:10.1371/journal.pone.0344624)
Supplement: Attachment — Submitted filename: Responses to Reviewers.docx [file pone.0344624.s004.docx]

**Multi-Trait Selection for Drought-Tolerant Soybean Genotypes Under Contrasting Water Regimes.**

**Authors’ Responses to Editor’s and Reviewers' Comments**

**Editor’s comments**

1. It is better to indicate that whether any genotype or genotypes among the genotypes is a known drought-tolerant genotypes or cultivars? What is the known drought-tolerant soybean reference cultivar?

**Response**: We thank you for this valuable comment. Although no known drought-tolerant cultivars were included, the accessions were chosen for their strong agronomic performance in Nigeria, including high yield potential, disease resistance and reduced pod shattering.

2. For your future research, I just suggest to follow a hill-dropping kind of sowing method to ensure the regular spacing between the genotypes.

**Response**: We sincerely appreciate your helpful suggestion. We will consider adopting a hill-dropping sowing method in future experiments to ensure more uniform spacing among genotypes.

3. Is it possible to have a durable abiotic stress resistance? Because some potential genotypes may not be having a consistency in performance and durability of resistance.

**Response**: We appreciate your insightful comment regarding the durability of abiotic stress resistance, and we will consider this point in future studies. In this study, we focused on intermittent drought tolerance because Nigerian farmers frequently face the “August break”, a short period of reduced rainfall that significantly affects soybean production, but there is a lack of information in the literature. Therefore, our study aimed to address this gap by evaluating accessions under conditions simulating this stress.

4. Have you had a thought of trying a superior genotype over existing varieties in cleistogamous species like *Glycine max* if you opt through plant breeding and selection?

**Response**: We appreciate this interesting suggestion. While soybean (*Glycine max*) is predominantly a self-pollinated (cleistogamous) species, opportunities exist to exploit superior genotypes through conventional breeding and selection. In our current study, the focus was on identifying superior accessions under contrasting water regimes. However, future breeding work will aim to integrate these superior genotypes into crossing programs to combine desirable traits and develop improved varieties adapted to local systems.

5. Dwarf varieties and their genetic, physiological mechanisms and relevant genes, like Rh1, Sd1, etc., were found to have an important role in the green revolution with yield in rice and wheat, for instance. Hence, have you tested any dwarf phenotype varieties? From Table 1, factorial loadings (FA1, FA2, and FA3) with the MGIDI index increase the yield (15.75) with SD: predicted selection gains. How do you infer or compare with reduced plant height if there is a case to study?

**Response**: Thank you very much for your question. Yes, dwarf varieties can be tested, and when included, MGIDI helps reveal whether shorter height contributes positively to yield gain. In our results, the 15.75% predicted selection gain for yield should be interpreted alongside the direction of selection for plant height. If yield increases while plant height decreases, the inference is consistent with the Green Revolution paradigm. If plant height reduction is not significant, then the genetic architecture of yield in our population may rely more on other adaptive traits rather than classic dwarfing genes like Sd1 or Rht1.

6) According to ANOVA from water stress and well-irrigated conditions, the plant height is not showing significance under the random effect of the environment, thus perhaps due to inherent variations in plant height with specific genotypes. Thus, I suggest selecting specific dwarf and tall genotypes to test with these water conditions to decipher yield and PH to compare further with your future studies.

**Response**: We thank you for this important remark. We acknowledged it and agree that including both dwarf and tall accessions in future evaluations under contrasting water regimes would provide valuable insights into the relationship between plant height and yield.

7) You have indicated in the abstract, "Pearson correlation analysis revealed that yields under stress and non-stress conditions showed a moderate positive correlation (r = 0.38), supporting stability across environments." However, it is expected that known drought-tolerant cultivars will have slightly more positive correlations than weak or medium correlations under water stress conditions as well, and it is possible to postulate the hypothesis of having drought-tolerant cultivars with reasonable yields as compared to high-yield lines. How do you infer this?

**Response**: Thank you for the insightful comment. The moderate positive correlation (r = 0.38) between yield under stress and non-stress conditions indicates that some genotypes maintained yield stability across environments, suggesting moderate drought tolerance. The correlation was not stronger likely because both tolerant and susceptible lines were included in the population. This supports the hypothesis that drought-tolerant cultivars can sustain reasonable yields under water stress, even if they do not have the highest yield potential. The result also reflects the known trade-off between yield potential and drought resilience.

According to Table 3, there is a huge difference between Yp: yield under well-watered conditions and Ys: yield under water-stressed conditions. But you have mentioned that 8 genotypes are consistently performing well in both conditions. What are all those 8 genotypes? Could you please compare them separately with an appropriate table or figure?

**Response**: Thank you for this meaningful remark. However, the eight genotypes mentioned were those commonly selected using the MGIDI approach from the trials conducted under both water-stressed and well-watered conditions. We have clarified this point in the Discussion section of the manuscript (P30 L620-L630).

Studied genotypes showing the higher broad-sense heritability in well-watered conditions over the stress conditions? Do you believe the genotypes would perform better after generations under stress conditions, as H2 is lower in drought conditions? You have also indicated that traits with high heritability (> 60%) indicate strong genetic control and a high possibility of reliable selection from the germplasm of interest.

**Response**: Thank you for the thoughtful comment. The higher heritability (H²) under well-watered conditions reflects reduced environmental influence, while the lower H² under drought indicates greater environmental variance. However, this does not preclude improvement under stress; continued selection in drought environments can still enhance genetic gain as adaptive alleles become fixed over generations. Thus, high H² under optimal conditions shows strong genetic control, whereas lower H² under stress highlights the complexity of drought tolerance rather than a lack of heritable variation.

The correlation figure and other figures have low resolution; please improve them. Please avoid overstating the predicted results, as you have also discussed the weakness of the MGIDI genotype method besides its strength.

**Response**: Thank you for your valuable feedback. We have replaced the correlation figure and other figures with higher-resolution versions to improve clarity. Additionally, we have carefully revised the relevant section to ensure that the interpretation of the predicted results is balanced, highlighting both strengths and limitations of the MGIDI method, as discussed in the manuscript.

**Response to Reviewer 1 comments**

**Summary**

The authors might add several sentences to clarify the scientific novelty of this study.

**Response**: We appreciate your valuable suggestion. In response, we added several sentences to the Introduction section of the manuscript to better highlight the scientific novelty and significance of this study.

**Main points**

1) P1 L2 (also P5 L102-104, and throughout the manuscript) Avoid using water stress and drought stress interchangeably. Drought is variation from climatic normality. Since your water deficit is applied artificially, it is better to use the term ‘water stress’, or ‘water deficit stress’ (to distinguish from flooding stress). Drought, from below average rainfall, is likely to have seasonal duration, and might vary from the short-term water deficits used in this study. Drought is also typically associated with other climatic factors such as higher temperature and/or lower humidity, as well as water deficit. In this study, short-term water deficit is a tool for determination of accession performance, and likely is related to drought tolerance, however, that was not measured in this study.

**Response**: Thank you for this insightful comment. We have revised the manuscript to consistently use “water deficit stress” or “water stress” instead of “drought stress, which we agree accurately reflects the controlled short-term conditions applied in this study.

2) P1 L3 title (also P1 L16, P1 L21, P3 L70, and throughout the document). Ensure that genotype is only used to describe a single genetic identity. Avoid the colloquial use of ‘genotype’ to describe a ‘line’, or ‘accession’. In most instance, ‘genotype’ can be replaced with ‘germplasm’. Table S1 in Supplementary materials describes the germplasm being used as ‘accessions’. Its not clear in this paper if the accessions were genotypes (i.e. inbred or clonal germplasm), but for soybeans, its likely these were selections from outcrossed germplasm, comprising many genotypes. Although there are precedents for colloquial use pf ‘genotype’ in the literature, “accessions” should not be referred to as ‘genotypes’, unless these are inbred lines comprising a single genotype.

**Response**: Thank you for this important clarification. We have thoroughly reviewed and revised the manuscript to ensure accurate and consistent terminology. The term “genotype” has been replaced with “germplasm” or “accession,” as appropriate, throughout the manuscript. This revision more accurately reflects the nature of the soybean materials used in this study, which comprised predominantly accessions rather than single, inbred genotypes.

3) P3 L48-49. Revise this sentence; as overly simplistic. Drought is infinitely variable, so giving a single yield loss is misleading. Drought can reduce soybean yield by 100%!!

**Response**: Thank you for this observation. We have revised the sentence to better reflect the variability and potential severity of water stress impacts (P3 L60- L62).

4) P4 L89. Describe the soil used for the study.

**Response**: We appreciate your observation. We have now included a description of the soil of our study area on P4 L101-L104 to provide better clarity on the experimental conditions.

5) P5 L100. Presumably, the replications were whole blocks?

Response: Thank for this observation. The experiment was laid out in an alpha-lattice design comprising 10 blocks per replication, with 15 accessions per block, and repeated across 3 replications. We have clarified this in the manuscript (P7 L157-160).

6) P5 L111. Clarify ‘Nodumax’. Was this a commercial product. Presumably is rhizobia?

**Response**: Thank you for this comment. Yes, Nodumax is a commercial soybean inoculant containing *Bradyrhizobium japonicum*, developed by IITA’s Business Incubation Platform, used to enhance nodulation and nitrogen in soybean. We have now clarified this in the manuscript (P9 L200-204).

**Specific points**

7) P1 L18 (also P5 L105). ‘Drought’ is a noun, and avoid colloquial use as an adjective. Suggest to replace ‘drought stress’ with ‘water stress’

**Response**: We thank you for this helpful suggestion. In response, we have replaced the term “drought stress” with “water stress” at P1 L20, P8 L183, and consistently throughout the manuscript.

8) P1 L78. Use past tense ‘aimed’. L79. Replace ‘are’, with ‘were’. L86. Replace ‘will provide’, with ‘provided’

**Response**: Thank you for this observation. We have revised the sentences accordingly, changing the verbs to the past tense as suggested: “aimed” (P6 L131), were (P6 L133).

9) P3 L56. Add ‘growth’, i.e. ‘… during critical growth stages…

**Response**: Thank you for the suggestion. We have revised the sentence at P3 L56 to include the word “growth” as suggested.

10) P4 L86. Use singular ‘gain’ (how many ‘genetic gains’ are you describing here?)

**Response**: We appreciate your observation. We have revised the wording at P22 L430 and throughout the manuscript, replacing ‘genetic gains’ with the singular form ‘genetic gain’.

11) P4 L96. Avoid the possessive term. Suggest “The climate of Ibadan is …”

**Response**: Thank you for the suggestion. We have revised the sentence at P7 L150-L151 to avoid the possessive form.

12) P5 L107. Avoid the possessive term. Suggest “plant”

**Response**: Thank you for the suggestion. We have revised the sentence at P3 L67 by replacing the possessive form with ‘plant’ as you suggested.

13) P5 L113. Replace “and that gives a total of”, with “equivalent to”

**Response**: We appreciate your suggestion. We have revised the sentence at P9 L205, replacing the expression ‘and that gives a total of’ with ‘equivalent to’ as suggested.

14) P5 L114-116. Use lower case ‘metribuzin, and ‘urea’. Check journal style, do these chemicals need their full chemical name at first mention?

**Response**: Thank you for this suggestion. We have corrected the capitalization at P9 L207 and L208, using lowercase for “metribuzin” and “urea”. We have also checked the journal guideline; however, the journal does not explicitly require full chemical names at first mention according to its online instructions.

15) P17 L313. Use singular ‘Multi-trait’

**Response**: Thank you for pointing this out. We have corrected the wording at P22 L413, changing “Multi-traits” to the singular form “Multi-trait” in line with your suggestion.

16) P17 L316 (also L411). Suggest to replace ‘Under’ with “During”

**Response**: Thank you for this helpful suggestion. We have replaced the wording at P22 L427, replacing “Under” with “During” in the manuscript.

17) P17 L322. Use the past tense ‘was’

**Response**: We appreciate this suggestion. We have revised the sentence at P24 L460, changing the verb to the past tense “was” as recommended.

18) P20 L389. Changes in what?

**Response**: Thank you for the observation. We have clarified the statement at P25 L492-L493 by specifying what the changes refer to, ensuring the context is clear and precise.

19) P20 L391. Delete ‘economic’. Drought will simply reduce yield, not need to include the term ‘economic’

**Response**: Thank you for the suggestion. We have revised the sentence at P25 L495 by removing the term ‘economic’ as water stress directly reduces yield without requiring this qualifier.

20) P20 L397. Replace ‘like’ with ‘such as’ (L438-439 is correct)

**Response**: Thank you for the suggestion. We have revised the sentence at P26 L505, replacing “like” with “such as”.

21) P23 L451. Use the past tense ‘indicated’

**Response**: We appreciate this suggestion. We have revised the sentence at P26 L550 to use the past tense ‘indicated’ as suggested.

**Response to Reviewer 2 comments**

(1) When different genotypes are planted in various regions with differing environments and soils, they may exhibit distinct characteristics. Therefore, field trials typically employ multi-point evaluations; however, it remains to be seen whether the drought-tolerant genotypes identified in one region can be applicable in other agro-ecological zones.

**Response**: We appreciate your insightful comment. We agree that genotype x environment interactions have roles in the expression of water deficit tolerance, and the tolerant accessions identified may perform differently across agro-ecological zones. In the present study, our objective was to conduct a first-step screening under controlled water deficit conditions in Ibadan, Nigeria. We have acknowledged this limitation in the manuscript (P32 L670-L673) and highlighted that subsequent multi-location trials across diverse environments and years will be essential to validate the stability and broad applicability of the tolerant accessions identified in the present study.

(2) While several methods were used to compare drought tolerance among genotypes, the study does not specify which method is more representative for assessing drought tolerance in soybeans.

**Response**: Thank you for this insightful comment. We acknowledge that multiple methods were used to compare water stress among the accessions. In the revised manuscript, we clarified that the MGIDI approach was considered the most representative and comprehensive method for assessing drought tolerance in soybeans, as it integrates multiple traits simultaneously to provide a balanced selection of superior accessions. In contrast, the drought tolerance indices selection method focuses primarily on yield-based performance.

(3) The manuscript is well-written and clear, but it could be further refined.

Response: We sincerely thank you for the positive feedback regarding the clarity of the manuscript. In response, we have carefully revised the manuscript to further enhance readability and ensure a clearer understanding.

**Response to Reviewer 3 comments**

**Title & Abstract**

1) The abstract is long and verbose. Consider tightening the language by focusing more on outcomes than method repetition

**Response**: We thank you for this valuable observation. We have revised the abstract to make it more concise, with reduced methodological details and greater emphasis on the main findings.

2) Mention how this research advances the field by demonstrating MGIDI's use for drought selection in tropical soybean germplasm.

**Response**: We appreciate this helpful observation. The abstract has been revised to emphasize how this study advances the field by applying MGIDI for drought tolerance selection in tropical soybean germplasm (P2 L33-L37).

**Introduction**
1) A clear articulation of the knowledge gap is lacking. What specifically is not known or has not been done in similar germplasm or environments?

**Response**: We thank you for this comment. We have revised the introduction (P4 L105- L116) to explicitly highlight the knowledge gap and how our study addresses it.

2) Please include information that clearly stating:

o Why previous selection strategies were insufficient.

**Response**: We appreciate this comment. We addressed the request in the manuscript (P4-P5, L78-L97) to provide further explanation of why previous selection strategies were insufficient.

o What novelty MGIDI + traditional indices bring in combination.

**Response**: We thank you for this comment. The introduction has been revised (P5-P6 L117-L121) to highlight the novelty of combining MGIDI with traditional drought indices.

o Why the IITA germplasm is uniquely suitable for this kind of selection.

**Response**: We appreciate this comment. We revised the manuscript (P6 L126- L130) to explain why the IITA germplasm is uniquely suitable for this analysis due to its broad diversity and adaptation to tropical environments.

3) A clear hypothesis is missing.

**Response**: We thank you for this comment. We have now specified a clear hypothesis in the manuscript (P6 L122-L125).

**Materials and Methods**

4) There is no control/check varieties. Without benchmark genotypes, it's difficult to interpret relative performance or derive conclusions about breeding progress

**Response**: We acknowledge this insightful comment. While no formal check varieties were included, evaluating 150 diverse accessions with MGIDI and drought indices enabled the identification of superior genotypes. However, such varieties will be included in our future multi-location studies.

5) Are the two-stage drought simulation realistic stress?

**Response**: We thank you for this valuable comment. The two-stage water stress simulation applied (14 days at 35 DAS and 7 days at 63 DAS) targets the flowering and pod-filling stages, which are the most drought-sensitive periods in soybean. Stress at flowering causes flower abortion and reduced pod initiation, while stress at the pod-filling stage decreases seed number and size, collectively leading to significant yield loss. This makes our simulation at these two stages both realistic and relevant. We have clarified this in the manuscript (P8 L190-L197).

6) Temperature and rainfall variations across the years/seasons are critical in drought studies.

**Response**: Thank you for your comment. We agree that variations in temperature and rainfall across years and seasons are critical in drought studies. These data have been provided in the manuscript (P7, L152- L167).

7) Add a climatic data figure comparing environmental conditions in WW and WS seasons.

**Response**: Thank you for the suggestion. In response, we added Figures S1 and S2, which present the monthly rainfall and temperature variation, respectively, under each water regime across the experimental period. These figures illustrate the environmental differences between the well-watered (WW) and water-stressed (WS) conditions, providing context for the observed responses.

8) The field management description lacks clarity on soil properties, nutrient status, and irrigation uniformity. These are critical for attributing differences in genotypic response to water regime rather than confounding effects.

**Response**: Thank you for highlighting the importance of providing detailed field management information. The soil properties of the IITA experimental station have been described in the manuscript (P7 L152-L155). Fertilization was applied uniformly according to recommended rates for soybean, and irrigation for the water-supplemented treatment was carefully managed to ensure consistent application across all plots. These measures help minimize potential confounding effects, ensuring that observed differences in genotypic performances can be attributed mainly to the imposed water regimes.

9) Two rows per plot is not experimentally viable

**Response**: We really appreciate your concern on this point. We used two rows per plot due to the large number of accessions (150 accessions) that had to be evaluated under each contrasting water regime within limited field space, and the accessions were randomized three times to minimized the field variation and improve the robustness of comparisons among accessions.

10) What was the kind of sprinkler irrigation system? Model? Etc.

**Response**: Thank you for the comment. In response, we have provided detailed information about the sprinkler irrigation system used in the experiment at P8 L186-L189.

11) It’s not clearly stated whether the experimental unit was plot-based or individual plant-based for statistical analysis.

**Response**: We appreciate this observation. As clarified in the revised manuscript (P9-P10 L214-L216) although some traits (e.g., plant height, pods per plant, seeds per pod) were measured on a subset of plants within each plot, their values were averaged to obtain a single plot mean. Thus, the experimental unit for all statistical analyses was the plot mean.

11) Clarify unit of replication and randomization protocol used within blocks. Was spatial variation corrected?

**Response**: We appreciate this valuable comment. We have addressed the concern by adding clarifying sentences in the revised manuscript (P7 L69-L176).

12) The rationale for timing and duration needs justification using prior physiological data on soybean water stress sensitivity. How did you choose these two time points?

**Response**: We thank you for this essential suggestion. We have added a justification for selecting these two stress periods in the revised manuscript (P8 L190-L197).

**Statistical Analysis**

13) While explained mathematically, lacks the MGIDI implementation explanation for breeders.

**Response**: Thank you for the comment. The MGIDI index was implemented using the *“metan”* R package following Olivoto and Nardino (2020). The process involved standardizing traits, performing factor analysis, defining an ideotype, and calculating each genotype’s distance from it. For breeders, MGIDI offers a practical multi-trait selection tool that identifies genotypes with balanced performance across yield and adaptive traits, simplifying selection decisions.

15) Include a statement on whether model assumptions were checked and met

Response: Thank you for the insightful comment. The model assumptions for MGIDI were verified before analysis. Normality (Shapiro–Wilk test), homogeneity of variances (Levene’s test), and multicollinearity (VIF) were all checked, and results confirmed that the data met the required assumptions, ensuring the reliability of the MGIDI results.

**Results and discussion**

16) Grain yield (GY) had low H² under both conditions; this warrants deeper discussion, especially since it's the main selection target. Is MGIDI offset low GY heritability? This contradicts genetic gain theory unless indirect selection traits are used.

**Response**: We thank you for this valuable point. We have revised the discussion to clarify that MGIDI leverages highly heritable secondary traits alongside yield, reducing the limitations of direct yield selection under variable environments (P28 L555-L571).

17) Ensure each figure clearly labels axes, index names, and genotype IDs.

**Response**: We appreciate this important suggestion. We have carefully revised all figures to ensure that axes are clearly labelled, index names are explicitly indicated, and genotype IDs are visible and easy to interpret.

18) Add a paragraph in discussion, comparing genotypes selected by MGIDI vs. traditional indices (beyond SY150). What kind of genotypes would have been missed without MGIDI?

**Response**: We thank you for this insightful comment. We added a paragraph in the discussion comparing accessions selected by MGIDI vs traditional indices. We also highlighted the accessions despite their good performance for most traits evaluated, which would have been missed without the MGIDI selection approaches (P31 L638-L648).

19) The discussion focuses heavily on confirming existing findings (e.g., GY drops with drought), but misses a chance to generalize implications for other tropical soybean breeding programs.

**Response**: We appreciate this insightful suggestion. We have revised the manuscript accordingly to address this request (P31-P32 L649-L653)

20) What can breeders take away from this dataset? How could this inform early-stage breeding or genomic selection?

**Response**: We thank you for this valuable suggestion. We revised the discussion to show how the dataset breeders can guide early-stage multi-trait selection and support genomic selection through MGIDI-based ideotype identification (P32 L654-660).

**Conclusion**

Slightly descriptive

**Response**: We appreciate this insightful feedback. To address this suggestion, the conclusion has been revised to be more analytical, emphasizing breeding implications, limitations, and future steps (P31 L662-L671).

Add a concise final paragraph with a call to action.

**Response**: We thank you for this helpful suggestion. We have added a concise final sentence with a clear call to action in the conclusion (P31 L671-L673)

No validation is provided for selected genotypes. Without validation, conclusions on stability remain speculative

**Response**: We thank you for this important observation. We have revised the conclusion to note that, without multi-location and multi-season testing, stability remains provisional and requires future validation.
