# Supplementary material for: Multi-trait selection for drought-tolerant soybean accessions under contrasting water regimes
Source: PLoS One. 2026 Apr 2;21(4):e0344624. doi: 10.1371/journal.pone.0344624 (PMC13046122; doi:10.1371/journal.pone.0344624)
Supplement: Attachment — Submitted filename: Review notes KOUAM (Ms. Ref. No. PONE-D-25-34303R1.docx [file pone.0344624.s005.docx]

**Reviewer Comments to Author:**

Ms. Ref. No.: PONE-D-25-34303R1

Ms. Full Title: Multi-Trait Selection for Drought-Tolerant Soybean Accessions Under Contrasting Water Regimes

The authors conducted a study looking at the performance and tolerance level of 150 soybean genotypes from IITA collection when exposed to drought. The study carried out across two growing seasons use multi-traits selection approach under both well-watered and water-stressed condition. This is an important study. However, clarifications and amendments are to be made before the paper to be considered for publication in this valued journal

**Methods:**

[1]. Provide the characteristics of the soil used for testing the tolerance. What is the field capacity (FC) of that soil? What is the Permanent wilting Point (PWP)?
[2]. You have to explain each formula and the meaning of each index. Does the increase of each of these indices is leading to the plant tolerance?
[3]. You stressed the plant twice: At 35 DAS for 14 days, corresponding to the flowering stage and at 63 DAS for 7 days, corresponding to the pod-filling stage. Before combining both stresses, using each stress independently would have allow the comparison the stress induced during the flowering stage and the one made during the pod filling stage and coming out with the most sensitive stage between the flowering and the pod filling phases. What did you not consider independent stress?

**Results:**

[1]. Are the results presented in Table 3 are for the 30 best performing genotypes under stress? It is the results for which year? Are they for the combined two years? as the experiment was carried out during two consecutive years
[2]. Results should also present the least performing genotypes under stress, the most sensitive accessions which also of significant importance for a breeder, who can use them for example in crossing when looking the type of gene action for the tolerance to drought

**Conclusion**

[1]. In the conclusion, It would be interesting to name the top (Example Top 5) genotypes tolerant to drought and also the top genotypes sensitive to drought
