# Supplementary material for: Multi-trait selection for drought-tolerant soybean accessions under contrasting water regimes
Source: PLoS One. 2026 Apr 2;21(4):e0344624. doi: 10.1371/journal.pone.0344624 (PMC13046122; doi:10.1371/journal.pone.0344624)
Supplement: Attachment — Submitted filename: Responses_to_Reviewers_auresp_2.docx [file pone.0344624.s006.docx]

**Multi-Trait Selection for Drought-Tolerant Soybean Genotypes Under Contrasting Water Regimes.**

**Authors’ Responses to Reviewers' Comments**

**Response to Reviewer 4 comments**

**Methods**

[1]. Provide the characteristics of the soil used for testing the tolerance. What is the field capacity (FC) of that soil? What is the Permanent Wilting Point (PWP)?

**Response**: We appreciate the reviewer's comment. Detailed information on the soil characteristics and soil water parameters, such as field capacity (FC) and permanent wilting point (PWP), has now been added to the methods section (L129-131).

[2]. You have to explain each formula and the meaning of each index. Does the increase of each of these indices is leading to the plant tolerance?

**Response**: We thank the reviewer for this valuable suggestion. All related details and explanations have now been provided in the Methods section (L212-219).

[3]. You stressed the plant twice: At 35 DAS for 14 days, corresponding to the flowering stage and at 63 DAS for 7 days, corresponding to the pod-filling stage. Before combining both stresses, using each stress independently would have allowed the comparison of the stress induced during the flowering stage and the one made during the pod filling stage and coming out with the most sensitive stage between the flowering and the pod filling phases. What did you not consider as independent stress?

**Response**: We thank the reviewer for this comment. While analyzing each stress separately could identify the more sensitive stage, our main objective was to evaluate overall performance under drought throughout the reproductive period. In addition, combining the two drought periods helps to stimulate real field conditions, where plants often experience water shortages at multiple stages.

**Results**:
[1]. Are the results presented in Table 3 for the 30 best-performing genotypes under stress? It is the results for which year? Are they for the combined two years? As the experiment was carried out during two consecutive years.

**Response**: We thank the reviewer for these questions. Yes, Table 3 shows the 30 best-performing genotypes under stress, based on their combined performance across the two years.

[2]. Results should also present the least-performing genotypes under stress and the most sensitive accessions, which are also of significant importance to a breeder, who can use them, for example, in crossing when looking for the type of gene action for tolerance to drought.

**Response**: We thank the reviewer for this valuable suggestion. This information is included in Supplementary Table S3, which presents the performance of all the genotypes, from the best- to the least-performing, along with their drought response indices.

**Conclusion**
[1]. In the conclusion, It would be interesting to name the top (Example Top 5) genotypes tolerant to drought and also the top genotypes sensitive to drought.

We thank the reviewer for this valuable suggestion. The Conclusion has been revised to explicitly identify the top five drought-tolerant genotypes and the top five drought-sensitive genotypes based on drought tolerance indices.
